# Supplementary figures and images for: Systemic interleukin 10 levels indicate advanced stages while interleukin 17A levels correlate with reduced survival in esophageal adenocarcinomas
Source: PLoS One. 2020 Apr 16;15(4):e0231833. doi: 10.1371/journal.pone.0231833 (PMC7162521; doi:10.1371/journal.pone.0231833)

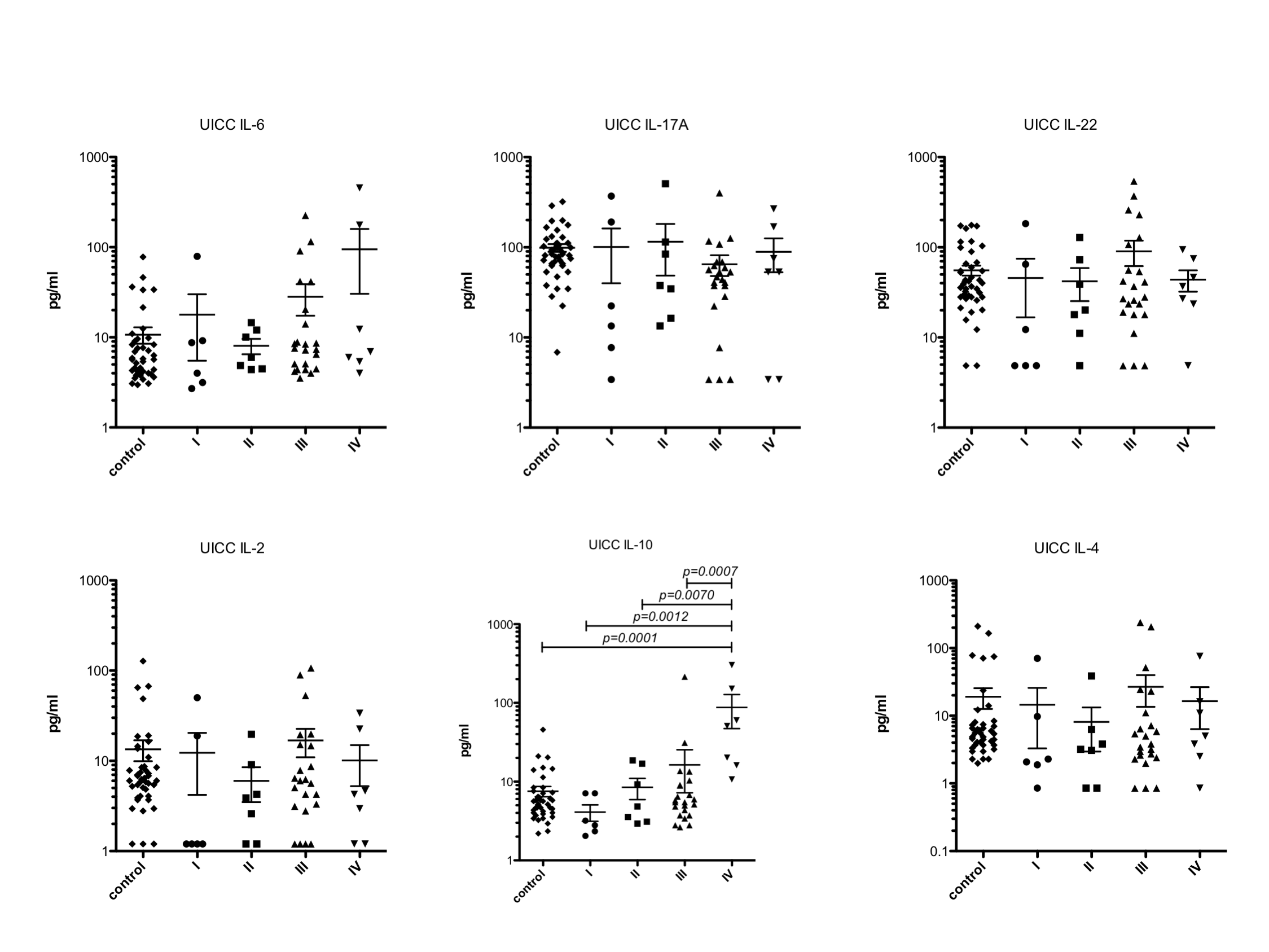

Supplement: S1 Fig — For better visualization p values are only given for significant tests (p<0.05). Bars indicate mean with standard error. (TIFF) [file pone.0231833.s001.tiff]
