# Supplementary material for: Systemic interleukin 10 levels indicate advanced stages while interleukin 17A levels correlate with reduced survival in esophageal adenocarcinomas
Source: PLoS One. 2020 Apr 16;15(4):e0231833. doi: 10.1371/journal.pone.0231833 (PMC7162521; doi:10.1371/journal.pone.0231833)
Supplement: S1 Table — T stage: describing the size and depth of tumor invasion; N stage: tumor involvement of nearby lymph nodes; M category: distant metastasis; UICC (Union for International Cancer Control): comprising the TNM categories; R: residual tumor at resection margins; G: grading of tumor cells. Significant p values (p<0.05) are highlighted in italic. Correlation coefficient is presented in parenthesizes. (DOCX) [file pone.0231833.s002.docx]

|  | **sex** | **age** | **T stage** | **N stage** | **M stage** | **G status** | **R status** | **UICC stage** | **BMI** | **deceased** |
| --- | --- | --- | --- | --- | --- | --- | --- | --- | --- | --- |
| **IL-6** | 0.761 (0.048) | 0.223 (-0.190) | 0.896 (0.021) | 0.503 (0.105) | 0.506 (0.104) | 0.805 (0.039) | 0.597 (-0.083) | 0.175 (0.211) | 0.506 (0.104) | 0.227 (0.188) |
| **IL-17A** | 0.345 (-0.147) | 0.262 (-0.175) | 0.445 (-0.120) | 0.286 (-0.167) | 0.697 (0.061) | 0.842 (0.031) | 0.561 (-0.091) | 0.978 (-0.004) | 0.686 (0.063) | 0.466 (-0.144) |
| **IL-22** | 0.742 (-0.052) | 0.159 (-0.219) | 0.445 (0.120) | 0.704 (0.060) | 0.685 (0.064) | 0.657 (0.070) | 0.254 (-0.178) | 0.283 (0.167) | 0.417 (0.127) | 0.653 (0.071) |
| **IL-2** | 0.794 (-0.049) | 0.264 (-0.174) | 0.717 (0.057) | 0.273 (0.171) | 0.857 (-0.028) | 0.436 (0.122) | 0.098 (-0.256) | 0.198 (0.200) | 0.663 (0.167) | 0.990 (0.002) |
| **IL-10** | 0.690 (-0.063) | 0.343 (-0.148) | *0.017 (0.362)* | 0.459 (0.116) | *<0.0001 (0.564)* | 0.718 (0.057) | 0.119 (0.241) | *0.003 (0.447)* | 0.786 (-0.043) | *0.008 (0.399)* |
| **IL-4** | 0.605 (-0.081) | 0.068 (-0.281) | 0.952 (0.009) | 0.932 (-0.013) | 0.485 (0.109) | 0.885 (-0.023) | 0.672 (-0.066) | 0.360 (0.143) | 0.550 (0.094) | 0.942 (-0.011) |
