# Supplementary material for: Systemic interleukin 10 levels indicate advanced stages while interleukin 17A levels correlate with reduced survival in esophageal adenocarcinomas
Source: PLoS One. 2020 Apr 16;15(4):e0231833. doi: 10.1371/journal.pone.0231833 (PMC7162521; doi:10.1371/journal.pone.0231833)
Supplement: Attachment — Submitted filename: Reply to reviewers.docx [file pone.0231833.s003.docx]

**Reply to reviewers**

ACADEMIC EDITOR:

1. The authors addressed that "Blood samples were collected from  the  biobank  of  the  Department  of  Surgery." (lines 109-110) The timing of blood drawing (at diagnosis, before treatment or during treatment, after treatment) in the EAC group has not been mentioned.

The editor is right about the lack of this information. All blood samples were drawn the day before surgery. To address this question we added the following sentence: “*Blood samples were drawn the day before surgery* *and immediately processed.*” (line 110-111; page 5)

2. The company, location, country information of experiment kits, and machines/devices are not clearly described.

We thank the editor for this point. We have added the company, location and the catalog number used for the analysis “*For serum cytokine quantification LEGENDplex^TM^ (BioLegend, Koblenz, Germany; Catalog no. 740721) human T helper cell cytokine panel was used.*” (line 126; page 5)

3. Multivariate analysis should include clinicopathological features to adjust their independent role.

We thank the editor for this remark. Indeed, we tested all cytokines against R status and UICC stage on multivariate analysis since both clinical parameters had the strongest influence on univariate survival analysis. However, we did not mention it properly within the manuscript. Hence, we added the following sentence to the method section: “*All cytokines were tested against UICC stage and R status in multivariate analysis.”* (line 141-142; page 6).

4. Lines 310-312, "However, a trend of increased levels of systemic IL-10 and decreased levels of IL-4 were found in EAC patients as compared to controls." But the p-value of IL-10 show near no significance. This sentence should be revised.

The editor is right about the missing significance for IL-10. To avoid misunderstandings when using the term „trend“, we changed the sentence accordingly: “*However, slightly, albeit not significantly, increased levels of systemic IL-10 and decreased levels of IL-4 were found in EAC patients as compared to controls.*” (line 312-315; page 14)

Reviewer #1: 1. The manuscript makes a contribution to the knowledge of systemic inflammatory interleukins in patients with esophageal adenocarcinoma.
2. Page 2 Line 42, the sentence “we wanted to investigated” should be “we wanted to investigate”.
3. May suggest English editing if necessary.

The grammar of the sentence was adjusted accordingly and the paper was further edited by a native English speaker.

Reviewer #2: I am not familiar with this area about relationship between EAC and IL. But I read this manuscript with interest. Please elaborate on why circulating IL-10 and IL-17A can help identify patients with advanced disease and may serve as potential targets for future tumor treatment.

We thank the reviewer for this positive comment, which will improve reading of the manuscript. We would like to further explain our findings. FOXP3^+^ regulatory T cells are one of the main producers of IL-10 and these cells are frequently found in different solid malignancies [1]. Recently, we were able to demonstrate a connection between increased FOXP3^+^ cells and IL-10 with survival in EACs on a local level. Interestingly, levels of IL-10 were not only associated with reduced survival in tumor tissue but also in the tissue without histological alterations distant to the tumor indicating a local field effect [2]. Hence, we hypothesized that IL-10 is not only increased on a local level but also secreted into the blood stream by FOXP3^+^ cells. Therefore, the level of systemic IL-10 might indicate an increased number of active FOXP3^+^ regulatory T cells. This is important since the activity of regulatory T cells could be targeted by check point inhibitor therapy, e.g. anti-CTLA4-mAb (ipilimumab) or anti-CCR4 leading to an oncologic benefit [3,4].

To further elaborate our findings we added the following passage to the discussion: ”*In a recent study, we reported an increased number of tissue resident IL-10 producing FOXP3^+^ regulatory T cells in EAC patients, which were significantly associated with reduced survival rates. Moreover, the correlation on patients’ survival was not only found for IL-10 within the tumor but also for the unaltered mucosa close to the resection margin representing a local immunological field effect [28]. Together with the current results, one might hypothesize, that this anti-inflammatory environment extends to the blood stream and helps malignant cells to evade host response and influences the oncogenetic and metastatic ability of neoplasms. Of note, IL-10 can have pleiotropic functions, which might depend on the cellular source. However, we suggest that manipulating regulatory T cells to produce less IL-10 might be a potential treatment option, which has already been tested in other malignancies with promising results* *[47,48].*” (line 333-344; page 14 and 15)

[1]	Takeuchi Y, Nishikawa H. Roles of regulatory T cells in cancer immunity. Int Immunol 2016;28:401–9. https://doi.org/10.1093/intimm/dxw025.

[2]	Karstens K-F, Kempski J, Giannou AD, Pelczar P, Steglich B, Steurer S, et al. Anti-inflammatory microenvironment of esophageal adenocarcinomas negatively impacts survival. Cancer Immunol Immunother CII 2020. https://doi.org/10.1007/s00262-020-02517-8.

[3]	Hodi FS, Butler M, Oble DA, Seiden MV, Haluska FG, Kruse A, et al. Immunologic and clinical effects of antibody blockade of cytotoxic T lymphocyte-associated antigen 4 in previously vaccinated cancer patients. Proc Natl Acad Sci U S A 2008;105:3005–10. https://doi.org/10.1073/pnas.0712237105.

[4]	Kurose K, Ohue Y, Wada H, Iida S, Ishida T, Kojima T, et al. Phase Ia Study of FoxP3+ CD4 Treg Depletion by Infusion of a Humanized Anti-CCR4 Antibody, KW-0761, in Cancer Patients. Clin Cancer Res Off J Am Assoc Cancer Res 2015;21:4327–36. https://doi.org/10.1158/1078-0432.CCR-15-0357.

Reviewer #3: This is an interesting proposal “Systemic interleukin 10 and 17A levels indicate advanced stages and reduced survival in esophageal adenocarcinomas” designed to evaluate the presence of systemic inflammatory interleukins (IL) and their impact on the prognosis. Of esophageal adenocarcinomas patient. The investigators found the significant higher systemic IL-6 and lower IL-17A in EAC patients and IL-10 levels has trend increase in EAC patients. There is a correlation between increases IL-10 with an increased T stage. Also, IL-10 levels were higher in patients with distant metastasis.

The investigators are doing measurement of Interleukins study in EAC patients with different conditions and need to provide clear data analysis to support their conclusion, such as survival issue.

We thank the reviewer for these remarks. It is true, that the enrolled EAC patients present with different clinical and tumor conditions. We evaluated the impact of these conditions on survival in univariate analysis (see Table 1). To further account for the variables with a significant influence on survival we performed a multivariate analysis including the UICC stage (comprising the TNM status) and R status with each cytokine. To clarify this approach, we added the following sentence to the method section: “*All cytokines were tested against UICC stage and R status in multivariate analysis.”* (line 141-142; page 6).

Also, to figure out the possible mechanism of IL10 as marker in EAC tumor, authors should provide the detail of the localization of IL10/17 and foxp3/CD25 in EAC with T stage or with distance metastasis.

The aim of this study was to characterize the systemic cytokines and investigate potential associations with clinicopathological parameters to evaluate potential cytokines for potential future use as biomarker. A detailed analysis of the origin of the cytokines (e.g. regulatory T cells, effector T cells) or the final localization of the latter T cells after circulating the blood stream is an interesting question and we have recently done part of this analysis. Indeed, we found that the amount of local expression of IL-10 by FOXP3^+^ cells is strongly associated with survival in EACs. We were also able to find a reduced amount of effector T cells (CD4^+^IL22^+^ and CD4^+^IL17^+^) in the local environment. To put our results in the right context, we have added the following passage to the introduction and discussion:

“*In addition, we recently demonstrated a strong association of IL-10 secreted by regulatory T cells with patients’ survival not only in the tumor tissue but also in the unaltered mucosa close to the resection margin representing a local immunological field effect [28]. On the basis of these results, we hypothesized that systemic IL-10 concentration might also correlate with the stage or survival of EACs. Therefore, the aim of this study was to measure systemic levels of IL-10*.” (line 82-88; page 3 and 4)

*”In a recent study, we reported an increased number of tissue resident IL-10 producing FOXP3^+^ regulatory T cells in EAC patients being significantly associated with reduced survival rates. Moreover, the impact on patients’ survival was not only found for IL-10 within the tumor tissue but also for the unaltered mucosa close to the resection margin representing a local immunological field effect [28]. Together with the results from the current study, one might hypothesize, that this anti-inflammatory environment extends to the blood stream and helps malignant cells to evade host response and influences the oncogenetic and metastatic ability of neoplasms. Of note, IL-10 can have pleiotropic functions, which might depend on the cellular source. Therefore, we suggest that manipulating regulatory T cells to produce less IL-10 might be a potential treatment option, which has already been tested in other malignancies with promising results [47,48*].*”* (line 333-344; page 14 and 15)

Other issues are listed as following:

Specific comments.

Major points

• Title: The title seems not appropriate. IL-10 level correlates TMN stage but not survival. IL-17 showed correlation with survival but seems not convincing enough.

The reviewer is right about the inaccuracy of the title. Hence, we changed the title of the manuscript to “*Systemic interleukin 10 levels indicate advanced stages while interleukin 17A levels correlate with reduced survival in esophageal adenocarcinomas*”.

• Line 57: It is not shown the correlation between IL-17 and advanced disease

The reviewer is right, that we did not identify a correlation between IL-17 and an advanced disease. Therefore, we changed the conclusion of the abstract as follows:” *In addition, circulating IL-10 might help to identify patients with advanced disease and IL-17A might indicate a limited prognosis.*” (line 55-57; page 2)

• 71: Rationales of selecting these 6 markers is unclear. Why other cytokines such as IL-8, IFNg are not selected?

Indeed, we also checked other cytokines (IL-5, IL-13, IL-9, IFNg, TNFa, IL-21 and IL-17F) but did not find significant changes in levels compared to controls (see Reply Figure 1) or any associations with clinical parameters (see Reply Table 1). Therefore, we focused on the cytokines we already had investigated on a local level (IL-17A, IL-22, IL-10). As additional pro-inflammatory markers we chose IL-2 and IL-6 and as an additional anti-inflammatory marker we chose IL-4 since interesting results have been shown for these cytokines in other studies for other entities. However, we are happy to include the other cytokines, as shown in the reply Figure below, if requested.

We also added the following part to the discussion: “*In addition, we recently demonstrated a strong association of IL-10 secreted by regulatory T cells with patients’ survival not only in the tumor tissue but also in the unaltered mucosa close to the resection margin representing a local immunological field effect [28]. On the basis of these results, we hypothesized that systemic IL-10 concentration might also correlate with the stage or survival of EACs. Therefore, the aim of this study was to measure systemic levels of IL-10*.” (line 82-88; page 3 and 4)

**Reply Figure 1: Comparison of cytokines in EAC patients and controls**

Significant differences (p<0.05) are highlighted in italic. Bars indicate mean with standard error.

**Reply Table 1: Spearman correlation matrix**

|  | **sex** | **age** | **T stage** | **N stage** | **M stage** | **G status** | **R status** | **UICC stage** | **BMI** | **deceased** |
| --- | --- | --- | --- | --- | --- | --- | --- | --- | --- | --- |
| **IL-5** | 0.673 (-0.066) | 0.113 (-0.245) | 0.819 (0.036) | 0.968 (-0.006) | 0.581 (0.086) | 0.762 (0.048) | 0.565 (-0.090) | 0.316 (0.156) | 0.795 (0.041) | 0.671 (-0.067) |
| **IL-13** | 0.907 (0.018) | 0.167 (-0.214) | 0.150 (0.223) | 0.116 (0.243) | 0.313 (0.157) | 0.452 (0.118) | 0.724 (0.055) | 0.240 (0.343) | 0.790 (0.042) | 0.772 (-0.046) |
| **IL-9** | 0.833 (-0.033) | 0.054 (-0.296) | 0.878 (-0.024) | 0.913 (-0.017) | 0.639 (0.074) | 0.790 (-0.042) | 0.365 (-0.142) | 0.569 (0.089) | 0.579 (0.087) | 0.932 (-0.013) |
| **IFNg** | 0.650 (0.071) | 0.123 (-0.239) | 0.609 (-0.080) | 0.794 (-0.041) | 0.201 (0.199) | 0.510 (-0.103) | 0.716 (-0.057) | 0.354 (0.145) | 0.691 (0.062) | 0.979 (0.004) |
| **TNFa** | 0.904 (0.019) | 0.078 (-0.272) | 0.733 (0.054) | 0.830 (0.034) | 0.395 (0.133) | 0.810 (0.038) | 0.671 (-0.067) | 0.216 (0.193) | 0.376 (0.138) | 0.852 (-0.029) |
| **IL-21** | 0.925 (-0.015) | 0.105 (-0.251) | 0.321 (0.155) | 0.983 (0.003) | 0.369 (0.141) | 0.501 (0.105) | 0.529 (-0.099) | 0.303 (0.161) | 0.243 (0.182) | 0.706 (-0.059) |
| **IL-17F** | 0.772 (0.046) | 0.167 (-0.215) | 0.669 (-0.067) | 0.761 (-0.048) | 0.412 (-0.128) | 0.910 (-0.018) | 0.206 (-0.197) | 0.819 (0.036) | 0.680 (0.065) | 0.699 (-0.061) |

T stage: describing the size and depth of tumor invasion; N stage: tumor involvement of nearby lymph nodes; M category: distant metastasis; UICC (Union for International Cancer Control): comprising the TNM categories; R: residual tumor at resection margins; G: grading of tumor cells. Significant p values (p<0.05) are highlighted in italic.

Correlation coefficient is presented in parenthesizes.

• 154: There is no trend in IL-10 level because p value is over 0.1
• 311: same as line 154, there is no trend of increase in IL-10
• 317: same as line 154, there is no trend of increase in IL-10

The reviewer is right about the missing significance for IL-10. To avoid misunderstandings when using the term „trend“ we changed the sentences accordingly: “*Slightly, albeit not significantly, elevated systemic IL-10 levels (p=0.265) with a median of 6.45 pg/ml (range 2.05 – 302.3) and reduced systemic…*” (line 155-156; page 7) and “*However, slightly, albeit not significantly, increased levels of systemic IL-10 and decreased levels of IL-4 were found in EAC patients as compared to controls.*” (line 312-315; page 14)

• 334: This might be saying too much. Data in this study shows a potential of IL-10 in diagnosis but it doesn't mean antagonizing IL-10 will have a therapeutic potential. IL-10 has been known to have pleiotropic function: anti-inflammatory effect and immunostimulatory effect. In the field of cancer therapy, pegylated IL-10 is used to induce cancer immunity that is opposite to antagonizing IL-10. Then it might be true that increase of IL-10 indicates increased inflammation caused by cancer but not necessarily means inhibition of IL-10 has a therapeutic effect.

The reviewer is right about the pleiotropic function of IL-10 and we have thus softened our conclusions: ”*Together with the current results, one might hypothesize, that this anti-inflammatory environment extends to the blood stream and helps malignant cells to evade host response and influences the oncogenetic and metastatic ability of neoplasms. Of note, IL-10 can have pleiotropic functions, which might depend on the cellular source. However, we suggest that manipulating regulatory T cells to produce less IL-10 might be a potential treatment option, which has already been tested in other malignancies with promising results [47,48]. However, a correlation with overall survival was neither found for systemic IL-10 nor systemic IL-4 in this study. To conclude, IL-10 demonstrated to be strongly associated with advanced stages of EACs but further investigations are needed to evaluate its influence on patient survival.*” (line 338-348; page 15)

• 369: I am not sure what the author is trying to say as a limitation.

With the mentioned paragraph, we wanted to remind the reader that analyses of systemic cytokines are inevitably influenced by multiple factors, which can’t be all taken into account. To provide a clearer statement we changed the paragraph as follows:” *A general limitation in analyzing systemic interleukins lies in the high number of confounding factors potentially influencing systemic interleukin levels. For example, Cook et al. recently described a potential link between excessive obesity and cigarette smoking with systemic inflammation [35]. Also, other environmental factors like alcohol or the composition of the microbiota might alter systemic inflammation. These limitations have to be kept in mind when interpreting our data. Another limitation is the lack of early stage esophageal carcinomas in the study. However, most of the patients with EACs are diagnosed at an already advanced stage due to the absence of symptoms.* “ (line 374-383; page 16)

• 376: same as line 154, there is no trend of increase in IL-10 in EAC patients

The reviewer is right about the missing significance for IL-10. To avoid misunderstandings when using the term “trend“ we changed the discussion to: “ *Importantly, systemic IL-10 levels correlate with the increase of the local tumor burden (T stage) and are also significantly elevated in patients with distant metastasis.*” (line 386-388; page 16 and 17)

• 380: IL-17 is not correlates with tumor stage so it could not be said
• 381: The data of this paper showed potential in diagnosis or prognosis rather than therapeutics

The reviewer is correct, that our findings rather support a diagnostic option than a therapeutic one and that IL-17 does not correlate with tumor stage. Therefore, we adjusted the final conclusion as follows:” However*, only IL-17A levels influenced survival in multivariate analysis. Hence, circulating IL-10 might help to identify EAC patients with advanced disease and systemic IL-17A might indicate a limited prognosis.*” (line 388-390; page 17) and the conclusion in the abstract to “*In addition, circulating IL-10 might help to identify patients with advanced disease and IL-17A might indicate a limited prognosis.*” (line 55-57; page 2)

Minor point
• According to Method, the sample used for assay is not serum but plasma but “serum” is used in the body.

We indeed used serum samples for the experiments. Hence, we changed the methods to “*Whole blood was centrifuged at 2000 rpm for 10 min. After centrifugation the total amount of serum from the 10 mL collection tube was transferred to another tube and stored at −80 °C until analysis.*” (line 123-125; page 5)
